# Evidence in dentistry guidelines

**DOI:** 10.1590/S1516-31802009000600005

**Published:** 2010-05-21

**Authors:** Cristiane Rufino Macedo, Álvaro Nagib Atallah

**Affiliations:** I DDS. Doctoral student, Universidade Federal de São Paulo - Escola Paulista de Medicina (Unifesp-EPM), São Paulo, Brazil.; II MD. Full professor and head of the Division of Emergency Medicine and Evidence-Based Medicine of Universidade Federal de São Paulo - Escola Paulista de Medicina (Unifesp-EPM). Director of the Brazilian Cochrane Center and Scientific Director of Associação Paulista de Medicina (APM), São Paulo, Brazil.

**Keywords:** Practice guideline, Dentistry, Oral medicine, Education, dental, Review [Publication Type], Guia de prática clínica, Odontologia, Medicina bucal, Educação em odontologia, Revisão [Tipo de Publicação]

## Abstract

**CONTEXT AND OBJECTIVE:**

Guidelines are suggestions for clinical practice based on the best available scientific evidence. Nevertheless, in drafting such guidelines, existing systematic reviews are often ignored and are replaced by general consensuses. This ends up compromising the quality of the instructions through bias. Our objective was to investigate whether Cochrane systematic reviews were present among the bibliographic references of prevention and treatment guidelines for dentistry that have been published in databases.

**DESIGN AND SETTING:**

This retrospective, observational study was conducted at the Brazilian Cochrane Center.

**METHODS:**

The databases were searched for guidelines. Any guidelines obtained were then checked to find whether Cochrane systematic reviews were present in the bibliographic references of the guidelines. In their absence, we checked whether such reviews had not been included because no reviews existed yet, or because such reviews had not been consulted despite already existing.

**RESULTS:**

223 studies were initially selected; of these, 77 were excluded. Of the 146 guidelines included, 46 could have made reference to existing systematic reviews, but only 13 studies did so. Among these 13 studies, eight were systematic reviews following Cochrane methodology. Thirty-three guidelines had not been drafted using published systematic reviews as references, and 100 guidelines had been unable to use Cochrane references because no reviews existed yet.

**CONCLUSION:**

It is necessary to increase awareness of the importance of using systematic reviews in drafting dentistry guidelines. Likewise, it is necessary to develop systematic reviews that answer questions on the various topics that remain unanswered.

## RESUMO

**CONTEXTO E OBJETIVO:**

Diretrizes são sugestões de condutas clínicas baseadas nas melhores evidências científicas disponíveis. No entanto, muitas vezes, para a construção da diretriz, as revisões sistemáticas existentes são desconsideradas e substituídas por consensos, o que acaba comprometendo a qualidade das suas instruções por serem enviesadas. O objetivo foi verificar a presença das revisões sistemáticas Cochrane nas referências bibliográficas das diretrizes de prevenção e tratamento na odontologia publicados nas bases de dados.

**LOCAL E TIPO DE ESTUDO:**

Estudo retrospectivo observacional, conduzido no Centro Cochrane do Brasil.

**MÉTODOS:**

Busca das diretrizes foi realizada nas bases de dados. A partir da obtenção delas, foram checadas a existência de revisões sistemáticas Cochrane nas suas referências bibliográficas. Na sua ausência, era verificada se a revisão sistemática não foi incluída porque ainda não existe ou porque, existindo, não foi consultada.

**RESULTADOS:**

Foram previamente selecionados 223 estudos, e destes, 77 foram excluídos. Das 146 diretrizes incluídas, 46 poderiam incluir em suas referências as revisões sistemáticas existentes, mas apenas 13 estudos o fizeram, dentre eles somente 8 eram revisões sistemáticas com metodologia Cochrane. Trinta e três diretrizes não usaram para a sua construção as referências de revisões sistemáticas já publicadas e em 100 delas não seria possível a inclusão das referências Cochrane por ainda não existirem as revisões.

CONCLUSÕES: É necessária a conscientização da importância do uso das revisões sistemáticas na construção de diretrizes em odontologia, bem como o desenvolvimento de revisões sistemáticas que respondam perguntas que versam sobre os diversos temas ainda não respondidos.

## INTRODUCTION

Many guidelines have been drafted in the field of dentistry, but much has been discussed about the true value of such guidelines. Thus, it can be asked whether these guidelines were drafted using the best available evidence. Guidelines are suggestions for clinical practice based on the best available scientific evidence and should be drawn up in a structured, sensible and honest manner. When faced with a lack of quality evidence, advice from specialists in the subject is used as the basis for clinical practice. Nevertheless, in drafting such guidelines, existing systematic reviews are often ignored and are replaced by general consensuses. This ends up compromising the quality of the instructions through bias. The objective of such guidelines is to guide professional conduct, so that clinical practice can be improved and the desired outcomes among patients can be achieved.^1^

The systematic development of guidelines with well-defined programs began in the late 1970s, when the US National Institutes of Health began to produce consensus statements. Throughout the 1980s, various organizations outside the US adopted these programs to develop their own consensus statements. Nevertheless, only in the 1990s did evidence-based medicine arise, thereby providing a meeting point for the best available evidence and clinical practice, which is fundamental for drawing up guidelines.^2^^,^^3^^,^^4^^,^^5^

It is important to consider three factors regarding the implementation and development of guidelines. The first concerns the formation of a team of experts for clinical judgment: the team must be interdisciplinary and comprise both generalists and specialists in the field for which the guidelines are being prepared. The second factor relates to taking patients’ opinions, expectations and values into consideration, in the light of the intervention to be recommended. Finally, the third factor comprises the search for the best clinical evidence available, which has been obtained from systematic research.^6^

Guidelines should be drafted based on a defined clinical problem so that the research question to be addressed is clear and precise in its objective and thus can guide the search for studies in the scientific literature. The recommendations should be widely supported by the greatest level of scientific evidence, based on locating and assessing scientific reviews on the topic in the literature that already exists. Systematic reviews seek to gather together all adequately conducted studies in order to respond to a given clinical question. After obtaining the primary studies, the ones included are assessed for methodological quality in order to identify clinical and statistical heterogeneity in the data and to assess potential risks of bias inherent to the way in which the study was implemented. A meta-analysis is then carried out in an attempt to integrate the studies included in the review. Since this type of analysis allows the sample size to be increased, it gives rise to a better chance of detecting results, when such results exist, and it reduces random effects and the level of uncertainty, as measured by the confidence interval. Hence, conclusions reached through a systematic review of the literature attain higher levels of evidence than other types of studies do.

## OBJECTIVE

The objective of this study was to investigate whether Cochrane systematic reviews were present among the bibliographic references of prevention and treatment guidelines for dentistry that have been published in electronic databases.

## METHODS

This retrospective, observational study was conducted at the Brazilian Cochrane Center, in the Universidade Federal de São Paulo - Escola Paulista de Medicina (Unifesp-EPM). This study included all published prevention and treatment guidelines for dentistry.

The outcome assessed was the presence of Cochrane systematic reviews in the bibliographic references of dentistry guidelines. In the absence of any reference to a Cochrane systematic review in the bibliography, we checked whether such reviews had not been included because no review existed yet, or because such reviews had not been consulted despite already existing.

### Search strategy for identifying studies

The search for guidelines was conducted in the following databases: Medical Literature Analysis and Retrieval System Online (Medline), from 1996 to March 2008; and Literatura Latino-Americana e do Caribe em Ciências da Saúde (Lilacs), from 1996 to March 2008.

The search strategy was developed based on medical subject headings (MeSH), using words referring to dentistry that were adapted to each database: dental general practice; dentistry; oral medicine; dentistry; stomatology; community dentistry; dental education; undergraduate dental education; and continuing dental education. The tool in each database was used to limit the search with regard to the type of publication (practice guidelines) and the date of publication (from 1996 to March 2008). Dentistry guidelines were selected when they met the inclusion criteria, i.e. when they concerned prevention or treatment; when they were in English, Spanish or Portuguese; and when publication was between 1996 and 2008.

### Data extraction

The search strategy identified relevant studies. The guidelines obtained were then checked to find whether Cochrane systematic reviews were present in the bibliographic references of the guidelines.

## RESULTS

Two hundred and twenty-three studies were initially selected; of these, 77 were excluded for the following reasons: they were not prevention or treatment guidelines; they were publication duplicates; there were publication errors in the reference; the language was not English, Spanish or Portuguese; or the full texts could not be read as was the case for eight studies.

**Figure 1.**
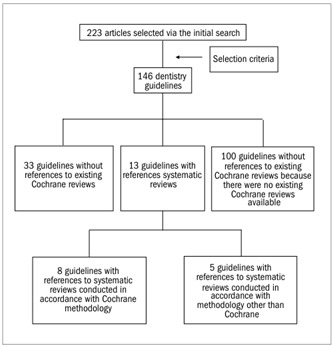
Results regarding presence of Cochrane systematic reviews in bibliographic references of prevention and treatment guidelines for dentistry.

Of the 146 remaining guidelines, 46 could have made reference to existing systematic reviews, but only 13 studies did so. Among these 13 studies, eight were systematic reviews following Cochrane methodology. Thirty-three guidelines had not been drafted using published systematic reviews as references, and 100 guidelines had been unable to use Cochrane references because no reviews existed yet (Figure 1).

## DISCUSSION

The results from this study demonstrate that for the majority of the guidelines, no concern was shown with regard to consulting the highest level of evidence at the time of drafting the guidelines. Of the 46 guidelines that could have included already-published systematic reviews in their conclusions, only 13 did so, while the other 33 guidelines did not use the available systematic reviews. Many guidelines included bibliographic references to books, overview studies, non-systematic reviews of the literature and consensuses reached by associations or societies to which the authors were connected.

It is interesting to note that, over the years, many points regarding certain types of conduct were decided upon in consensuses and were subsequently named “guidelines”. It would be expected that, at some point during this process, the conclusions from systematic reviews would be included, but this does not coincide with our findings, since 80% of the guidelines did not make reference to Cochrane systematic reviews that were already available on the subject matter. One example of this is the 2006 guidelines on the use of fluoride for cavity prevention, in which a systematic review on this topic in 2002 was completely ignored.^7^^,^^8^

The lack of systematic reviews in the bibliographies cannot be explained by difficulty in accessing them, given that systematic reviews are available in primary databases (e.g. Medline and the Cochrane Systematic Review Database). It is possible that failures to include systematic reviews among the references have been due to lack of knowledge regarding the importance of including this type of study in guidelines.

Through data interpretation, systematic reviews allow the weight of evidence to be judged, along with the applicability of such evidence to clinical practice. When such reviews do not exist, it is important to find primary studies (e.g. clinical trials, cohort studies or case-control studies), so that a synthesis of the evidence can be produced for inclusion in the guidelines. Thus, recommendations based on the best level of evidence, can be categorized as strong or weak, according to whether studies with appropriate results and quality assessments for responding to each clinical question have been included.^9^^,^^10^

One observational study evaluated the attributes of guidelines that influenced decision-making by professionals in their clinical practice.^11^ It was concluded that evidence-based recommendations are followed more frequently by health professionals than are recommendations not based on the best scientific evidence.^11^ Thus, guidelines that are developed by multidisciplinary teams, using knowledge produced by systematic reviews, with a high degree of recommendation and strength of evidence, can furnish professionals with clear evidence to guide them in making decisions.^12^^,^^13^^,^^14^

Guidelines based on specialists’ opinions or on non-systematic research are widely criticized for failing to reflect the present state of knowledge in healthcare. Guidelines drafted without well-defined criteria are of limited value, because they give rise to high risk of bias.^12^ Currently, many guidelines are still developed by one or another isolated, single-specialty group that can be compared to a situation of “good old boys sat around the table” (GOBSAT), who base their recommendations on their individual knowledge and not on evidence. These recommendations are thus far more susceptible to bias and conflict of interest.^3^ Moreover, such guidelines can be considered to be a waste of time and money, given that drawing them up is both time consuming and costly.

The Appraisal of Guidelines Research and Evaluation (AGREE), published in 2001, was the first instrument for assessing guideline quality that could be used to draft guidelines. Guidelines need to be planned in accordance with a structured program, and coordinated under the rubric of evidence-based, guideline-development principles in order to ensure high quality.^4^^,^^15^ Moreover, guidelines should be updated as new evidence is published.^2^

Nevertheless, there is still a great need for systematic reviews in the field of dentistry, even before new guidelines can be published. The production of systematic reviews in dentistry by the Cochrane Collaboration began in 1996 with the creation of the Oral Health Group, but only after 2000 did the publication of new reviews become more intense. To date, the Cochrane Collaboration has conducted 84 systematic reviews on dentistry; nevertheless, many questions regarding treatment and prevention remain unanswered, given the gaps between systematic reviews in dentistry.

## CONCLUSION

The majority of guidelines in dentistry do not take Cochrane systematic reviews into consideration, given that they do not make reference to them in their bibliographies or conclusions. It is necessary to increase awareness of the importance of using systematic reviews in drafting guidelines, so that recommendations are based on the best evidence available in dentistry.

There is a need for new systematic reviews that answer questions on the various topics that remain unanswered, so that these can also be included in the guidelines.

It is important to note that the Cochrane Library is accessible free of charge in many countries, including in Brazil since 2001, through www.centrocochranedobrasil.org.
